# Role of arachidonic acid metabolism in intervertebral disc degeneration: identification of potential biomarkers and therapeutic targets via multi-omics analysis and artificial intelligence strategies

**DOI:** 10.1186/s12944-023-01962-5

**Published:** 2023-11-25

**Authors:** Jianye Tan, Meiling Shi, Bin Li, Yuan Liu, Shengzhong Luo, Xigao Cheng

**Affiliations:** 1 Department of Orthopaedics, The Second Affiliated Hospital of Nanchang University, Nanchang, 330006 China; 2 Jiangxi Key Laboratory of Intervertebral Disc Disease, Nanchang University, Nanchang, Jiangxi 330006 China; 3 Institute of Orthopedics of Jiangxi Province, Nanchang, 330006 Jiangxi China; 4 Institute of Minimally Invasive Orthopedics, Nanchang University, Jiangxi, 330006 China; 5 Medical College of Nanchang University, Nanchang, 330006 China

**Keywords:** Intervertebral disc degeneration, Machine learning, Artificial neural network, Biomarkers, Single-cell sequencing, Arachidonic acid

## Abstract

**Background:**

Intervertebral disc degeneration (IVDD) is widely recognized as the primary etiological factor underlying low back pain, often necessitating surgical intervention as the sole recourse in severe cases. The metabolic pathway of arachidonic acid (AA), a pivotal regulator of inflammatory responses, influences the development and progression of IVDD.

**Methods:**

Initially, a comparative analysis was conducted to investigate the relationship between AA expression patterns and different stages of IVDD using single-cell sequencing (scRNA-seq) data. Additionally, three machine learning methods (LASSO, random forest, and support vector machine recursive feature elimination) were employed to identify hub genes associated with IVDD. Subsequently, a novel artificial intelligence prediction model was developed for IVDD based on an artificial neural network algorithm and validated using an independent dataset. The identified hub genes were further subjected to functional enrichment, immune infiltration, and Connectivity Map analysis. Moreover, external validation was performed using flow cytometry and real-time reverse transcription polymerase chain reaction analysis.

**Results:**

Both scRNA-seq and bulk RNA-seq data revealed a positive correlation between the severity of IVDD and the AA metabolic pathway. They also revealed increased AA metabolic activity in macrophages and neutrophils, as well as enhanced intercellular communication with nucleus pulposus cells. Utilizing advanced machine learning algorithms, five hub genes (*AKR1C3*, *ALOX5*, *CYP2B6*, *EPHX2*, and *PLB1*) were identified, and an incipient diagnostic model was developed with an AUC of 0.961 in the training cohort and 0.72 in the validation cohort. An in-depth exploration of the functionality of these hub genes revealed their notable association with inflammatory responses and immune cell infiltration. Lastly, AH6809 was found to delay IVDD by inhibiting *AKR1C3*.

**Conclusions:**

This study offers comprehensive insights into potential biomarkers and small molecules associated with the early pathogenesis of IVDD. The identified biomarkers and the developed integrated diagnostic model hold great promise in predicting the onset of early IVDD. AH6809 was established as a therapeutic target for *AKR1C3* in the treatment of IVDD, as evidenced by computer simulations and biological experiments.

**Supplementary Information:**

The online version contains supplementary material available at 10.1186/s12944-023-01962-5.

## Introduction

Intervertebral disc degeneration (IVDD) is a prevalent condition that significantly contributes to global disability, particularly by causing debilitating low back pain [1, 2]. The intervertebral disc comprises the nucleus pulposus (NP), annulus fibrosus, and cartilaginous endplates [3]. NP cells and the extracellular matrix (ECM) collectively maintain the normal structure and function of the intervertebral disc [4, 5]. Disruption of the delicate balance in NP and ECM homeostasis plays a pivotal role in the pathogenesis of IVDD, often resulting in an inflammatory cascade [6]. The activity of the arachidonic acid (AA) metabolic pathway and the mediation of downstream lipid products trigger inflammatory reactions, leading to an inflammatory storm. To address this issue, the study focus was directed towards the metabolic pathways of AA, a pivotal regulatory factor in the inflammatory response influencing the development and progression of IVDD.

At present, nonsteroidal anti-inflammatory drugs (NSAIDs) are widely employed to mitigate pain and inflammation in IVDD [7]. These drugs function by impeding AA metabolism, thereby diminishing the production of inflammatory mediators [8]. However, the utility of NSAIDs is constrained by their potential adverse effects, emphasizing the imperative for alternative approaches that specifically address the aberrant AA metabolic pathway associated with IVDD.

The diagnosis of IVDD primarily relies on imaging studies such as magnetic resonance imaging (MRI) [9]. MRI enables the assessment of intervertebral disc hydration, disc height, and alterations in disc contour, thereby facilitating IVDD diagnosis. However, quantifying these changes during the initial stages of IVDD presents considerable challenges. Notably, only patients experiencing severe back pain actively seek MRI examinations, impeding the early detection of IVDD. Moreover, MRI possesses certain limitations, including high costs and prolonged examination durations. Additionally, the clinical utility of MRI is constrained by specific contraindications, such as patients with pacemakers, intrauterine devices, pregnant women, or individuals with claustrophobia, who are ineligible for MRI examinations [10]. Current clinical management strategies for IVDD primarily emphasize disease progression mitigation, often culminating in surgical interventions as a last resort [7, 11]. However, these approaches do not adequately address the urgent need for early detection and personalized therapeutic interventions, which could alleviate the burden on both patients and healthcare systems [12, 13].

The identification of biomarkers associated with AA metabolism bears significant clinical relevance. Employing machine learning algorithms, such as LASSO (Least absolute shrinkage and selection operator), random forest (RF), support vector machine recursive feature elimination (SVM-RFE), and artificial neural networks (ANNs), facilitates the screening and selection of biomarkers indicative of AA pathway dysregulation [14]. These biomarkers can serve as valuable clinical tools for early diagnosis, risk stratification, and predicting therapeutic responses, ultimately enhancing personalized treatment strategies for individuals with IVDD.

This study aimed to investigate the clinical significance of biomarkers associated with AA metabolism in the context of IVDD. Through the application of machine learning algorithms, this study to elucidate the intricate connection between AA metabolism and IVDD, thereby making a substantive contribution to the progress of precision medicine in this field. The objective was to identify dependable biomarkers and pioneer innovative diagnostic and therapeutic strategies, with the overarching goal of transforming the clinical management of IVDD and enhancing the well-being of patients with IVDD.

## Materials and methods

### Data collection and preprocessing

The keywords “intervertebral disc,” “degeneration,” and “human” were employed to query relevant datasets in the Gene Expression Database (GEO) (https://www.ncbi.nlm.nih.gov/geo/). Following a meticulous screening process, four datasets (GSE176205, GSE205535, GSE67567, and GSE70362) were selected for subsequent analysis. Notably, GSE205535 was a single-cell RNA sequencing (scRNA-seq) dataset, while the remaining datasets consisted of bulk RNA-seq data. For the three bulk RNA-seq datasets (GSE176205, GSE67567, and GSE70362), the "SVA" package was employed to mitigate batch effects. IVDD with grades ranging from normal to I–III were classified as mild IVDD, whereas discs with a grade of IV or higher were classified as severe IVDD. The 62 AA genes are provided in Supplementary file S1.

### scRNA-seq analysis

The “Seurat” R package was utilized for processing the scRNA-seq data and generating Seurat objects. The data was normalized and scaled accordingly. To filter the cells, the following exclusion criteria were applied using the “Seurat” R package: 1) Cells expressing less than 200 genes were excluded. 2) Cells expressing more than 6,000 genes were excluded. 3) Cells with mitochondrial gene content greater than 20% were excluded. Following the application of these criteria, a total of 9,161 cells remained for subsequent analysis. For dimensional clustering, the number of principal components (PCs) was configured to 18. To integrate the samples and mitigate batch effects, the “harmony” function was employed. Unsupervised cell clustering was accomplished using a graph-based method, leveraging the top 18 PCs. Visualization of the cell clusters was performed through UMAP plots. The Wilcoxon rank-sum test algorithm was employed to identify marker genes for each cell cluster, utilizing the “FindAllMarkers” function. Marker genes were selected based on the following criteria: 1) logFC greater than 0.25. 2) *P*-value less than 0.05. 3) min.pct greater than 0.1. In addition, the “AddModuleScore” function was utilized to compute the score of AA metabolism pathways within each re-clustered nucleus pulposus cell (NPC). Based on quartiles of the AA metabolic score of NPCs, the cells were categorized into three groups: high, medium, and low AA metabolic activity.

### Cell–cell communication analysis

The “CellChat” and “Seurat” packages were integrated to conduct cell–cell communication analysis [15]. Following data filtration, scRNA-seq data were processed using “Seurat.” Next, “CellChat” was employed to quantify communication pathways, compute information flow, and identify specific pathways within cell types of interest. In the investigation of cell–cell communication across various cellular contexts of AA metabolism, a comprehensive analysis focused on microglial cells was conducted to ascertain the strength of communication in each signaling pathway. Furthermore, particular communication pathways were selected for subsequent visualization.

### Pseudotime analysis of single-cell

The dynamic states of NPCs were assessed using the Monocle algorithm (version 2.18.0) for pseudotime analysis [16]. Monocle employs an unsupervised algorithm to order single-cell whole-transcriptome profiles, generating a developmental trajectory that depicts the progression of individual cells during differentiation. The "reduce Dimension" function was employed to compute the "CellDataSet" object, thereby reducing the dimensionality of the trajectory. To achieve effective dimension reduction and discrimination between data points, the discriminative dimensionality reduction based on trees method was chosen. Following dimension reduction, the two most informative features were extracted and utilized as coordinate axes to visualize the trajectory. To elucidate the mechanisms underlying fate decisions, branched expression analysis modeling was conducted to identify genes displaying branch-dependent expression patterns.

### Machine learning-based analysis of bulk RNA-seq

The LASSO method was employed for dimensionality reduction in the analysis of high-dimensional data. Specifically, the LASSO algorithm [17] was implemented using the "glmnet" package in R. In this analysis, the response type was configured as binomial, and the alpha parameter was set to 1. Employing LASSO regression, a penalty function aimed at refining the model was developed by reducing the number of variables while preserving the most valuable ones. To ascertain the optimal penalty parameter (λ), tenfold cross-validation was conducted, enabling the identification of the λ value corresponding to the lowest cross-validation error.

Furthermore, the RF method was employed, utilizing the “randomForest” package in R, to identify significant disease-specific genes [18]. Initially, the optimal number of trees in the RF model was determined through the evaluation of the minimum cross-validation error. This error was computed by comparing discrepancies between two sets of samples and the overall error across all samples. Next, the RF model was constructed, and gene significance was gauged using the Gini coefficient method, facilitating the acquisition of dimension importance values. This method assesses model precision by reducing the Gini coefficient. Employing this approach, the top 11 disease-specific genes with the highest importance scores were identified.

Finally, the SVM-RFE machine learning strategy was employed to identify hub genes [19]. In this approach, the “SVM” package in R was utilized to train an SVM model on the training dataset, followed by sorting the features based on their weights. Subsequently, a stepwise feature elimination process was initiated, whereby the least significant features were iteratively removed, and the SVM model was retrained after each elimination until the desired number of features was achieved. The optimal number of features was determined through cross-validation, and the performance of the SVM models was assessed using metrics such as accuracy or area under the receiver operating characteristic (ROC) curve.

### Construction of a neural network to develop a disease classification model

The R software package “NeuralNet” (version 1.44.2) was employed to construct the input layer of the ANN model, utilizing genes acquired from the RF screening [20]. The model parameters consisted of five hidden layers within the ANN. The classification model for IVDD was developed by incorporating information regarding gene weights. In this model, the disease classification score was determined as the product of the weight score and the gene expression level, as illustrated below.

$${\varvec{n}}{\varvec{e}}{\varvec{u}}{\varvec{r}}{\varvec{a}}{\varvec{H}}{\varvec{F}}=\sum (Gene\hspace{0.17em}expression\times Neural\hspace{0.17em}network\hspace{0.17em}weight)$$

The confusion matrix function was employed for cross-validation to assess the model's accuracy, and the “pROC” package was utilized to estimate the AUC for evaluating the classification performance [21]. Subsequently, a nomogram was constructed using the "rms" package in R, incorporating the hub genes associated with IVDD.

### Gene set variation analysis (GSVA)

Utilizing the R package “GSVA” (version 2.11), GSVA was performed to examine disparities in enriched gene sets among various subtypes. The database files from three sources, namely KEGG, Reactome, and WikiPathways, were extracted from the MSigDB website (https://www.gsea-msigdb.org/gsea/msigdb) for GSVA. Differential biological functions and pathways were identified by comparing GSVA scores between subtypes, employing the “limma” R package (version 3.52.1) [22].

### Gene set enrichment analysis (GSEA)

The reference gene sets were derived from the MSigDB. Differential expression analysis was performed via GSEA to elucidate the impact of the genome on biological functions.

### Evaluation of infiltrating cells in IVDD

To investigate disparities in immune cell infiltration between the two subtypes, multiple methods and software tools were employed, including CIBERSORT-ABS, CIBERSORT, EPIC, MCPCOUNTER, QUANTISEQ, TIMER, and XCELL, to assess immune cell infiltration levels in patients with IVDD [23–28].

### Friends analysis

The method defines functional similarity as the geometric mean of Gene Ontology (GO) semantic similarity for molecular function and cellular components. This calculation incorporates both the protein's functional role and its cellular localization. The GO semantic similarity is computed utilizing the "GOSemSim" package, and the resultant functional similarity serves as a quantitative measure indicating the strength of the association between proteins [29]. This approach can be employed to pinpoint crucial genes and significant biomarkers in a given pathway.

### Screening small-molecule drugs

In contrast to conventional small-molecule drug screening approaches, the eXtreme Sum (XSum) method was employed to discern prospective small-molecule drugs for addressing disc degeneration [30]. Initially, the molecular characteristics of drugs were obtained from the Connectivity Map database. Subsequently, candidate small-molecule drugs were analyzed using the XSum algorithm, focusing on hub genes implicated in the process of IVDD.

### Molecular docking

Molecular docking analysis was conducted as described previously. The 3D structures of the proteins aldo–keto reductase family 1 member C3 (AKR1C3), cytochrome P450 family 2 subfamily B member 6 (CYP2B6), epoxide hydrolase 2 (EPHX2), and arachidonate 5-lipoxygenase (ALOX5) were retrieved from the Protein Data Bank (https://www.rcsb.org/) with the corresponding protein IDs: 4DBS, 4RQL, 6HGV, and 7TTJ, respectively. The 3D structure of the phospholipase B1 (PLB1) protein was predicted using AlphaFold. The ligand structure, AH6809 (PubChem CID: 119,461), was acquired from the PubChem database (https://pubchem.ncbi.nlm.nih.gov/) and subsequently converted into a 3D structure using Open Babel 2.3.1. It was subjected to subsequent energy minimization employing the MMFF94 force field. The highest-scoring docking results were visualized and analyzed using PyMOL.

### Cell lines

The immortalized human NPC line was acquired from ScienCell Research Laboratories, Inc. These cells were cultivated in Nucleus Pulposus Cell Medium (ScienCell Research Laboratories, Carlsbad, CA, USA) supplemented with 10% fetal bovine serum and 1% penicillin/streptomycin. The cells were maintained in a 37 °C incubator with a 5% CO_2_ atmosphere. NPCs exposed to either 100 μM tert-butyl hydroperoxide (TBHP) (Invitrogen, Carlsbag, CA) or 10 μM AH6809 (Sigma-Aldrich, St-Louis, MO) were utilized as an in vitro model for NPC degeneration.

### Real-time reverse transcription polymerase chain reaction (RT-qPCR)

Total RNA was extracted from NPC lysates using the AG RNAex Pro reagent (Accurate Biotechnology Co., Ltd., Hunan, China). Subsequently, 500 ng of total RNA was reverse transcribed into cDNA employing the Evo M-MLV RT Kit (Accurate Biotechnology Co., Ltd., Hunan, China) as previously described [31, 32]. RT-qPCR analysis was performed using the SYBR Green Premix Pro Taq HS qPCR Kit (Accurate Biotechnology Co., Ltd., Hunan, China) on the Bio-Rad CFX Connect System (Bio-Rad, CA, USA). Results were calculated using relative expression levels and the 2-^ΔΔ^Cq method. Primer sequences are presented in Table 1.

**Table 1:** Primer sequences for RT-qPCR in this study

Gene	Forward primer (5’ to 3’)	Reverse primer (5’ to 3’)
AKR1C3	GTCATCCGTATTTCAACCGGAG	CCACCCATCGTTTGTCTCGTT
MMP-3	AGTCTTCCAATCCTACTGTTGCT	TCCCCGTCACCTCCAATCC
ADAMTS5	GAACATCGACCAACTCTACTCCG	CAATGCCCACCGAACCATCT
ACAN	ACTCTGGGTTTTCGTGACTCT	ACACTCAGCGAGTTGTCATGG
COL2A1	TGGACGATCAGGCGAAACC	GCTGCGGATGCTCTCAATCT
COX2	CTGGCGCTCAGCCATACAG	CGCACTTATACTGGTCAAATCCC
GAPDH	CTCCAAAATCAAGTGGGGCG	TGGTTCACACCCATGACGAA

### Cell transfection

Cells were transfected with an overexpression plasmid during the logarithmic growth phase. Plasmid transfection was conducted using EndoFectin™ Max (GeneCopoeia, Guangzhou, China) following the manufacturer's instructions. Briefly, the transfection reagent was added to 125 µL of Opti-MEM (Gibco, Carlsbad, California, USA) containing the plasmid and incubated for 5 min. Subsequently, the mixture was left undisturbed for 15 min. The culture medium was replaced after 8 h, and cell harvesting was performed 24 h following plasmid transfection, as previously described [31].

### Apoptosis analysis

The alterations in apoptosis subsequent to transfection were assessed as follows: Cells were digested using pancreatic enzymes, excluding EDTA, and subsequently centrifuged at 300 × g for 5 min to separate the medium. Apoptosis was quantified utilizing flow cytometry, employing the Annexin V-FITC Apoptosis Assay Kit (BestBio, Shanghai, China) according to the manufacturer’s protocol. Data analysis was performed using FlowJo software (version 10; FlowJo LLC).

### Statistical analyses

All statistical data analysis and graph plotting were conducted using R software (version 4.1.1). To assess disparities between two groups, the Wilcoxon test was employed. Meanwhile, a one-way ANOVA was employed to ascertain any statistically significant differences among multiple groups. The threshold for establishing statistical significance was set at a *P*-value of < 0.05.

## Results

### ScRNA-seq profiling revealed heterogeneity in IVDD progression

**Fig. 1:**
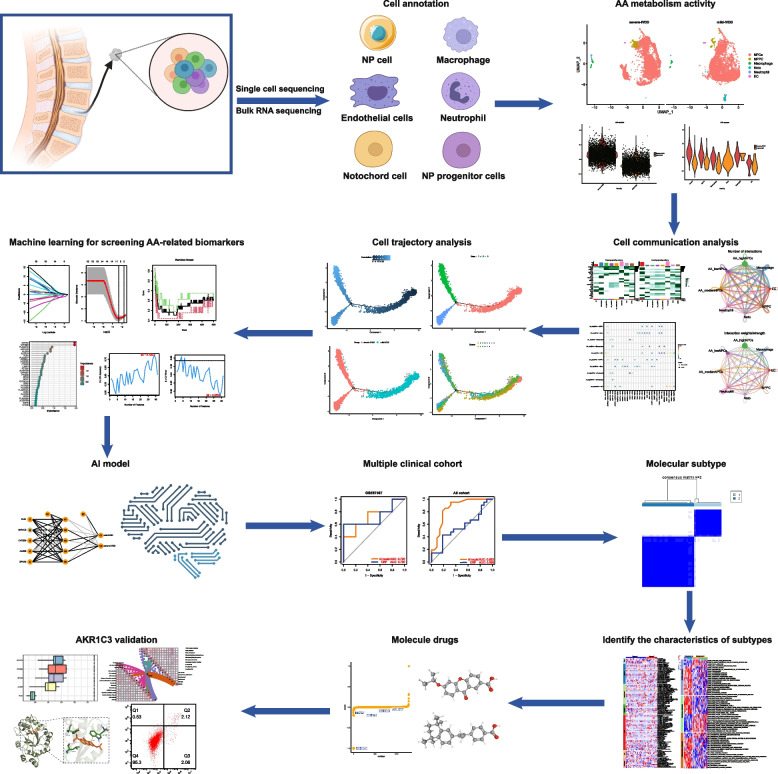
Flowchart of the study

**Fig. 2:**
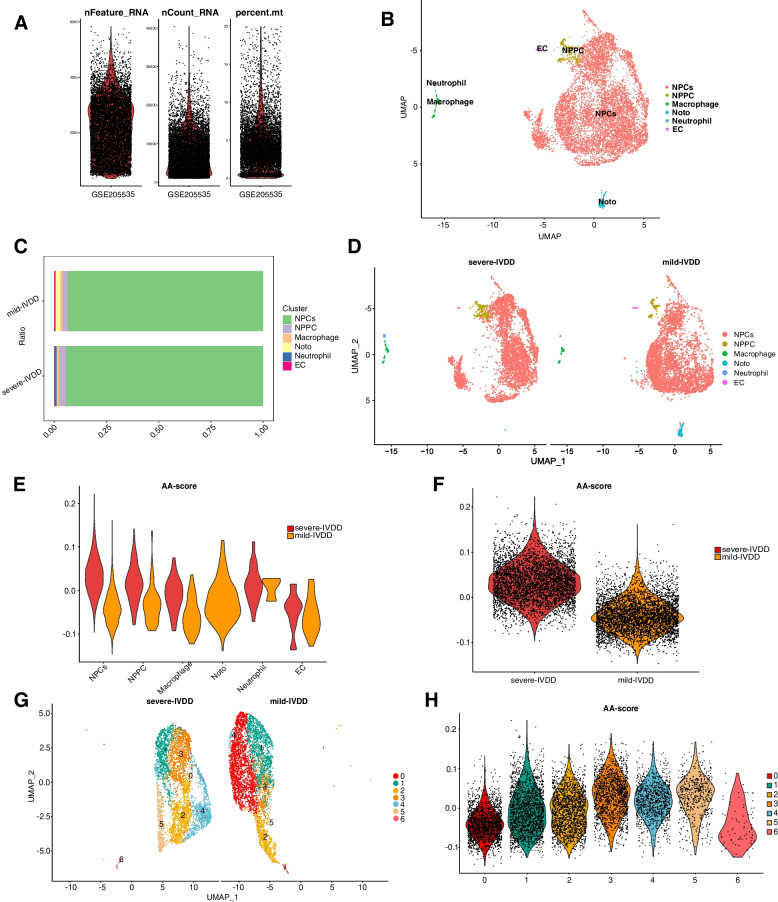
Enrichment analysis of hub genes in IVDD. **A** Following standard quality control on all cells pertaining to mild and severe IVDD, 9,161 cells were included in the analysis. **B** A UMAP plot illustrates six distinct cell types within the dataset, as identified through unsupervised clustering (NPCs, nucleus pulposus cells; NPPCs, nucleus pulposus progenitor cells; Noto, notochord cells; EC, erythroid cells). Each color represents a specific cell type. **C** Proportions of different cell types in mild-IVDD and severe-IVDD samples. **D** UMAP plots depict the distribution of AA metabolic activity in each cell. **E**, **F** A violin plot displays the AA metabolic score of various cell types in mild IVDD and severe IVDD. **G**, **H** A violin plot displays the AA metabolic score of NPCs in mild IVDD and severe IVDD

Figure 1 illustrates the flow of this study. Initially, scRNA-seq data pertaining to a single IVDD sample obtained from the GEO database was utilized to elucidate the intrinsic heterogeneity underlying the degenerative process in IVDD. After rigorous quality control procedures, cells exhibiting suboptimal quality were excluded, resulting in the selection of 9,161 cells for subsequent analysis (Fig. 2A). Each cell exhibited a mitochondrial UMI rate below 10%, and a notable correlation was observed between the number of detected genes and sequencing depth. Employing a principled approach, PCA-based dimensionality reduction was performed with a resolution value set to 1, identifying a total of 18 distinct cell clusters. These clusters exhibited pronounced heterogeneity, reflecting diverse cellular states within the intervertebral disc. Leveraging marker genes reported in prior studies, comprehensive cell type annotation was performed, and six categories of cells were characterized, including NPCs, nucleus pulposus progenitor cells (NPPC), macrophages, notochord cells (Noto), neutrophils, and endothelial cells (ECs) (Fig. 2B). Furthermore, proportional analysis of different cell types in the samples indicated that NPCs occupied more than 90% of the NP tissue, while the proportion of Noto in severe IVDD was lower compared to that in mild IVDD (Fig. 2C).

### Dynamic modulation of AA metabolism during NPC degeneration

Significant variations were observed in AA metabolic pathway activity across various stages of IVDD, with a positive correlation observed between higher degenerative degrees and increased AA metabolic pathway activity (Fig. 2D). Additionally, among all cell types examined, NPCs exhibited the most pronounced fluctuations in AA metabolic pathway activity, demonstrating elevated levels in cells characterized by greater degeneration (Fig. 2E, F). Therefore, NPCs were further investigated in this study. Subsequently, transcriptomic data pertaining to NPCs were extracted, and a reanalysis was conducted employing UMAP visualization (Fig. 2G). To assess AA metabolic pathway activity in each cell cluster of NPCs, the "AddModuleScore" function was utilized. The results are presented in Fig. 2H. Cluster 3 displayed the highest activity, while Cluster 0 exhibited the lowest activity.

### Cell communication analysis of NPCs exhibiting varying AA metabolic activity: unveiling the intricate interplay

**Fig. 3:**
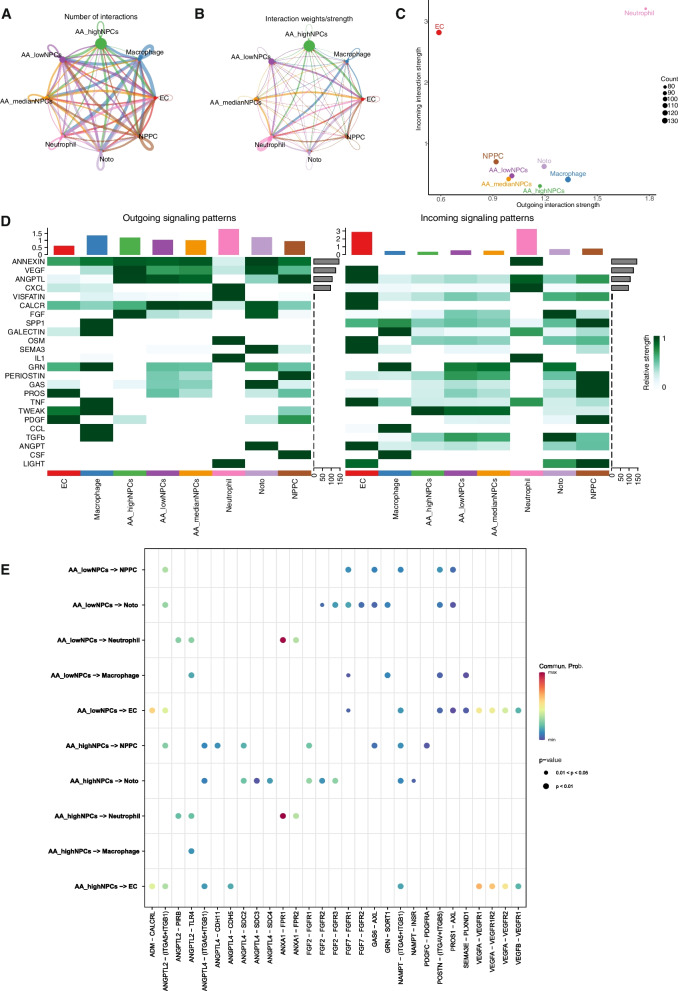
Diagram illustrating the intercellular communication network among NPCs exhibiting varying AA metabolic activity and other co-localized cell types. **A**, **B** Diagram depicting interactions between NPCs of varying AA metabolic activity and other cell types. The thickness of the connecting lines between two cell types indicates their interaction weight or intensity. **C** Point plots of outgoing and incoming signal pathways in various cell types. **D** Heatmap depicting the intensity of intercellular interactions among eight distinct cell type© (**E**). Summary of LR interactions between NPCs of varying AA metabolic activity and other cell types. The horizontal axis represents ligand cells and their corresponding receptor cells, while the vertical axis represents different signaling pairs

To elucidate the impact of AA on the microenvironment of the intervertebral disc, the differences in intercellular communication across different cell types were analyzed utilizing the “CellChat” R package. NPCs were stratified into three distinct categories based on quartiles (25% and 75%) derived from prior assessments; specifically, NPCs were categorized as exhibiting high, medium, or low AA metabolic activity. The construction of the intercellular communication network involved the aggregation of interaction frequencies and their corresponding weights. Figure 3A and B provide a visual representation of the strength of interactions in both incoming and outgoing signal pathways, thus emphasizing the central and intricate role that NPCs play in intercellular communication. Notably, NPCs with high AA metabolic activity exhibited elevated signal strength in outgoing pathways compared to the other two categories of NPCs, whereas NPCs with low AA metabolic activity exhibited heightened activity in incoming signal pathways (Fig. 3C, D). Moreover, a subsequent analysis, focused on ligand–receptor (LR) pairs, delved into the reciprocal communication patterns between NPCs of the aforementioned three categories and other cell types. NPCs with high AA metabolic activity demonstrated the ability to engage in cellular communication with NPPCs through ANGPTL4–(ITGA5 + ITGB1), ANGPTL4–CDH11, ANGPTL4–SDC2, FGF2–FGR1, and PDGFC–PDGFRA interactions; with EC through ANGPTL4–(ITGA5 + ITGB1) and ANGPTL4–CDH5 interactions; and with Noto via ANGPTL4–(ITGA5 + ITGB1), ANGPTL4–SDC2, ANGPTL4–SDC3, ANGPTL4–SDC4, FGF2–FGFR1, NAMPT–(ITGA5 + ITGB1), and NAMPT–INSR interactions. Additionally, NPCs with low AA metabolic activity engaged in cellular communication with NPPCs through FGF7–FGFR1, POSTN–(ITGAV + IGTB5), and PROS–AXL interactions; with Noto via ANGPTL2–(ITGA5 + ITGB1), FGF7–FGFR1, FGF7–FGFR2, GAS6–AXL, GRN–SORT1, POSTN–(ITGAV + ITGB5), and PROS1–AXL interactions; with macrophages through FGF7–FGFR1, GRN–SORT1, POSTN–(ITGAV + ITGB5), and SEMA3E–PLXND1 interactions; and with ECs through FGF7–FGFR1, POSTN–(ITGAV + IGTB5), PROS–AXL, and SEMA3E–PLXND1 interactions (Fig. 3E). These findings highlight the potential significance of AA metabolic pathway activity in NPCs for their communication with various cell types through these receptor interactions.

**Fig. 4:**
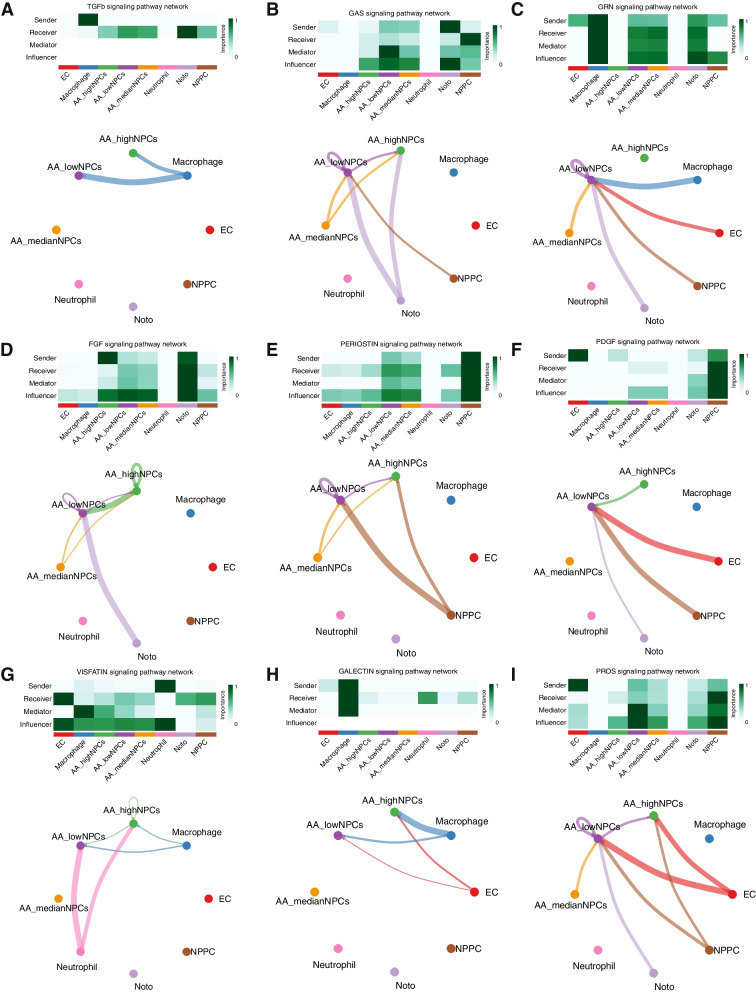
Circos diagram illustrates the interrelationships between NPCs exhibiting varying AA metabolic activity and other cell types across various signaling pathways. A heatmap showcases the relative likelihood of NPCs exhibiting varying AA metabolic types playing four distinct roles (sender, receiver, mediator, and influencer) within the signaling pathways. The color intensity corresponds to the magnitude of cellular impact

Analyzing the interactions between NPCs and other cell types via LR analysis revealed that these interactions were closely associated with several cellular processes, including cell proliferation, apoptosis, oxidative stress, ECM formation, and lectin response. This study revealed that within the TGFβ signaling pathway, NPCs of diverse AA metabolic types function as receptors that are influenced to varying degrees by macrophages (Fig. 4A). In signaling pathways linked to cell proliferation, NPCs of distinct AA metabolic types serve multiple roles as recipients, mediators, and influencers. They are also influenced by other cell types, with particularly strong associations observed between NPCs exhibiting low AA metabolism (Fig. 4B, C). Regarding processes involving the ECM, fibroblast differentiation signaling pathways, and lectin response signaling pathways, NPCs characterized by low AA metabolism assume more active roles as influencers (Fig. 4D–F). In contrast, within stress signaling pathways, NPCs with high AA metabolism function were observed to be more potent mediators (Fig. 4G–I). Therefore, it is evident that NPCs with different AA metabolic types play pivotal roles in regulating the onset and progression of IVDD by modulating the immune microenvironment.

### Pseudotime analysis revealed alterations in NPC metabolic activity in response to varying AA metabolic activity

**Fig. 5:**
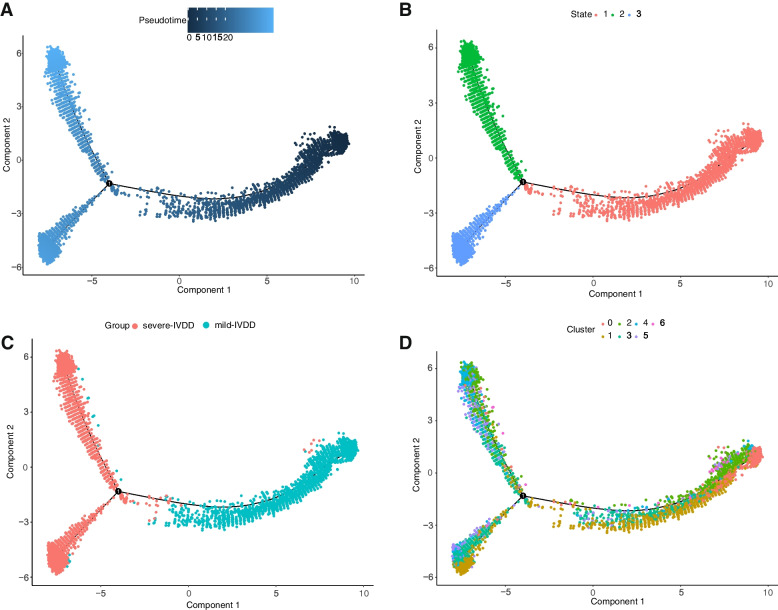
Pseudotime trajectory analysis of NPC clusters. **A** Pseudotime trajectory differentiation plot of NPCs. **B** Pseudotime trajectory depicting distinct differentiation states of ©s. **C** Differentiation of NPCs in mild IVDD and severe IVDD. **D** Pseudotime trajectory differentiation plot based on different clusters of NPCs

To further investigate the cellular trajectories of distinct clusters of NPCs involved in AA metabolism, the Monocle pseudotime algorithm for pseudotime analysis was employed, as shown in Fig. 5A, where various NPC clusters are distinguished by different colors. In this pseudotime analysis, shades of blue denote the temporal progression of cell differentiation, with darker shades signifying earlier stages of differentiation. The results presented in Fig. 5B demonstrate that NPCs underwent differentiation from right to left over time, with the lightest shade of blue corresponding to the most recently differentiated cell state 1, while cell states 2 and 3 represent later stages of NPC differentiation. Figure 5C illustrates the distribution of early-stage degenerated NPCs and late-stage degenerated NPCs during the differentiation process, while Fig. 5D showcases the distribution of distinct subgroups of NPCs along the cellular differentiation trajectory. By examining the variations in AA metabolic activity among these subgroups, Cluster 1, characterized by the lowest metabolic activity, was determined to primarily reside in the early stages of the cellular trajectory, corresponding to early degenerated NP tissue. Conversely, Cluster 3, exhibiting the highest metabolic activity, predominantly occupied the later stages of the cellular trajectory, indicative of late-stage degenerated NP tissue. These findings robustly support the close association between AA metabolic activity and IVDD progression.

### Screening hub genes via machine learning algorithms

**Fig. 6:**
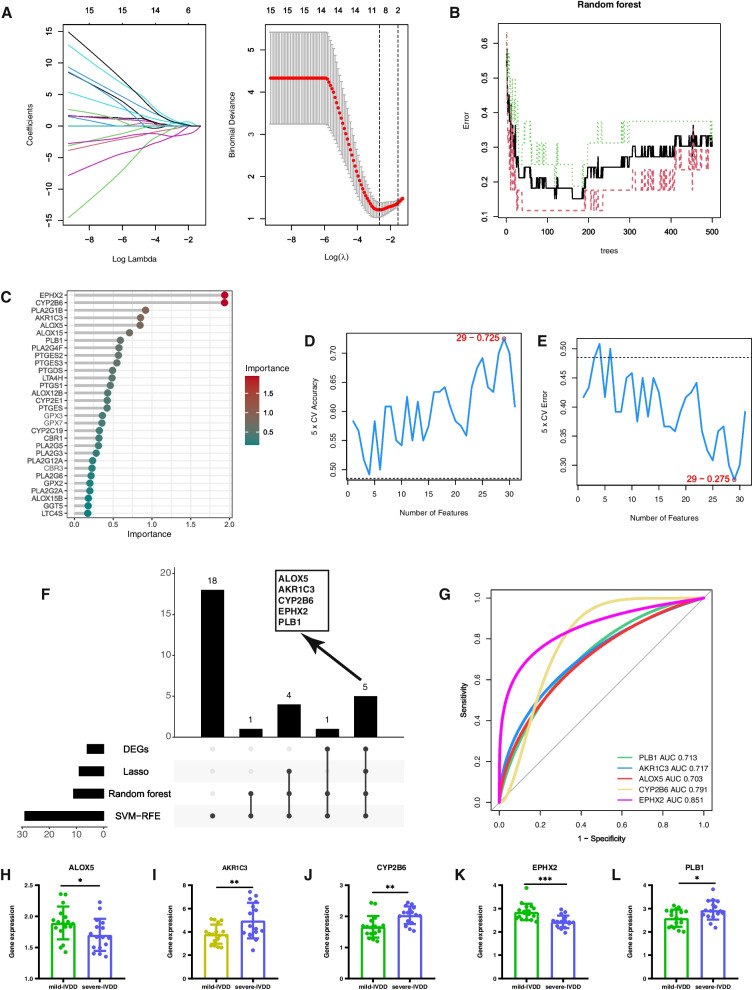
Development of the early diagnosis model for IVDD. **A** ROC curves of SVM and RF models. **B** The impact of the number of decision trees on the error rate. The x-axis represents the number of decision trees, and the y-axis indicates the error rate. The error rate stabilized at approximately 500 decision trees. **C** Results of the RF classifier's Gini coefficient approach. Genetic variation is on the x-axis, and the significance index is on the y-axis. **D**, **E** Gene selection process using SVM-RFE and tenfold cross-validation in the GSE70362 and GSE176205 datasets. The highest model accuracy was achieved when 29 genes were selected. **F** UpSet plot showcasing the characteristic genes in LASSO, RF, DEGs, and SVM-RFE. **G** ROC curve values for the five hub genes in the GSE70362 and GSE176205 datasets. **H**–**L** Representative bar graphs reveal the expression differences in ALOX5, AKR1C3, CYP2B6, EPHX2, and PLB1 between mild IVDD (*n* = 17) and severe IVDD (*n* = 16)

Three well-validated machine learning algorithms, namely LASSO, SVM-RFE, and RF, were employed to identify essential biomarkers associated with IVDD in datasets GSE70362 and GSE176205. The LASSO algorithm was subjected to rigorous tenfold cross-validation to ensure the robustness of the findings. On the basis of the optimal lambda value (0.07000546), the most influential features were judiciously selected to construct the LASSO model, leading to the identification of nine pivotal genes (*PLA2G1B*, *PLA2G4F*, *PLB1*, *PTGDS*, *AKR1C3*, *ALOX5*, *CYP2B6*, *EPHX2*, and *ALOX15*) (Fig. 6A). Additionally, the RF approach identified 12 significant genes (*AKR1C3*, *ALOX15*, *ALOX5*, *CYP2B6*, *EPHX2*, *LTA4H*, *PLA2G1B*, *PLA2G4F*, *PLB1*, *PTGDS*, *PTGES2*, and *PTGES3*) (Fig. 6B, C). Employing the SVM-RFE algorithm, the classifier was observed to achieve the highest accuracy and a commendable AUC value when employing a set of 29 optimal feature genes. Noteworthy among these genes were *PTGDS*, *AKR1C3*, *PLB1*, *ALOX5*, *ALOX15*, *EPHX2*, *PLA2G1B*, *GGT5*, *CYP2E1*, *ALOX12B*, *PTGES2*, *PLA2G4F*, *CYP2B6*, *CBR1*, *TBXAS1*, *LTC4S*, *PLA2G12A*, *GPX3*, *GPX7*, *LTA4H*, *PTGS1*, *PLA2G6*, *ALOX15B*, *PLA2G5*, *CBR3*, *PTGES3*, *CYP2C19*, *PTGES*, and *PLA2G3* (Fig. 6D, E). After obtaining the aforementioned machine learning results, the R package was utilized to generate plots depicting five hub genes (*AKR1C3*, *ALOX5*, *CYP2B6*, *EPHX2*, and *PLB1*) based on machine learning-filtered hub genes and differentially expressed genes (DEGs) (Fig. 6F). Figure 6G also demonstrates that individual hub genes exhibited precision in distinguishing different stages of IVDD (all predicted probabilities were greater than 0.7). Figures 6H–L depict the differential expression of these five hub genes in the NP tissue of IVDD. Among them, *AKR1C3*, *CYP2B6*, and *PLB1* showed high expression in severe IVDD, whereas the opposite expression pattern was observed for the other genes.

### Development of the AI model

**Fig. 7:**
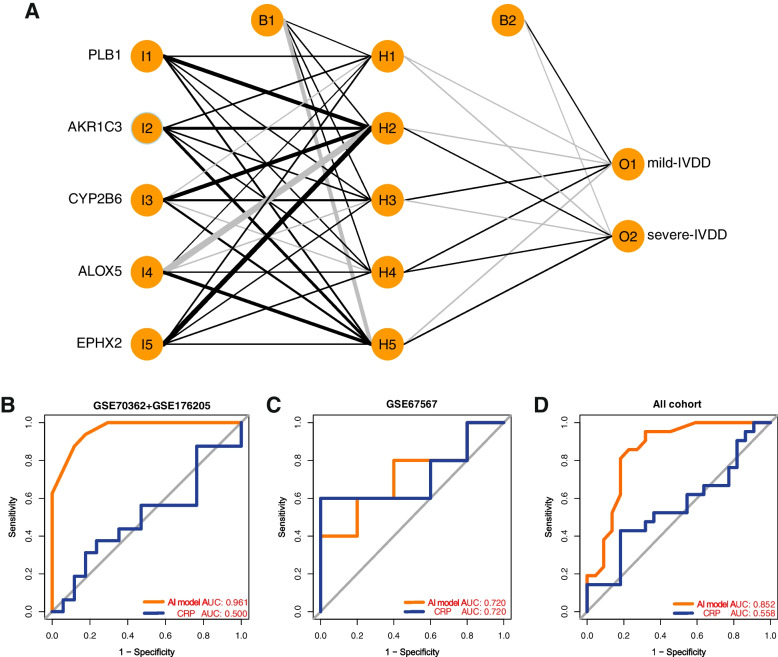
Development of the early diagnosis AI model for IVDD. **A**. The AI-ANN structure consists of five convolution layers, three max-pooling layers, and two fully connected layers. **B** AUC validation in the GSE70362 and GSE176205 datasets. **C** AUC validation in the GSE67567 dataset. **D** AUC validation in all datasets (GSE70362, GSE176205, and GSE67567)

To validate the classification potential of the hub genes, an ANN was initially utilized to construct an AI prediction model using the GSE70362 and GSE176205 datasets. The primary objective was to demonstrate the predictive capability of the hub genes for the early diagnosis of IVDD. In the R "neuralnet" package, the ANN architecture comprised five input layers, five hidden layers, and two output layers. Specifically, through the computation of the gene weights, optimal discrimination between mild and severe disc degeneration was achieved (Fig. 7A). Subsequently, ROC curves were generated to evaluate prediction accuracy. The AUC values approached 1 (AUC = 0.961), indicating the model's robustness. To assess the diagnostic model's performance, validation was performed using the test dataset (GSE67567), which yielded an AUC of 0.720. The AUC across all datasets, including both training (GSE70362 and GSE176205) and validation (GSE67567) datasets, amounted to 0.852. Furthermore, as depicted in Fig. 7B–D, the diagnostic efficacy of the AI model in discriminating between mild IVDD and severe IVDD substantially outperformed that of C-reactive protein (CRP) [33]. Thus, AI models exhibited significant diagnostic value within the entire dataset, enabling more precise assessment of inflammation levels and effective differentiation between various stages of IVDD.

### IVDD classification based on AA-related hub genes

**Fig. 8:**
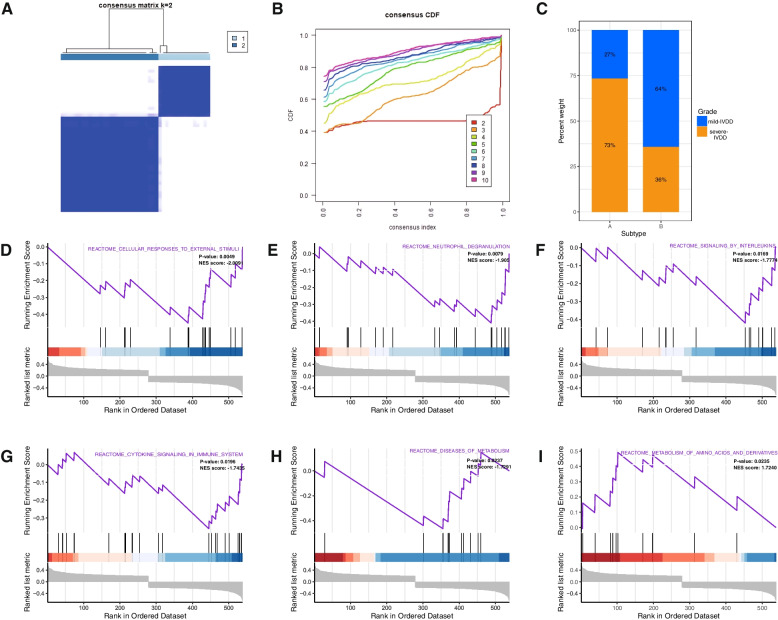
Identification of two different IVDD subtypes based on five hub genes. **A** Unsupervised clustering based on hub gene expression and consensus matrices for k = 2. **B** Overall CDF curves. **C** A bar chart illustrates the proportions of mild-IVDD and severe-IVDD groups in the two subtypes. **D**–**I** GSEA of Subtype A and Subtype B

To further investigate the relationship between disc degeneration and hub genes, an analysis of the expression profiles of hub genes in 43 IVDD samples was conducted using k-means unsupervised clustering. The most stable number of subtypes was determined to be two (k = 2) (Fig. 8A). Subsequently, based on the CDF curves, the 43 IVDD samples were clustered into two distinct subgroups: Subtype A (*n* = 15) and Subtype B (*n* = 28) (Fig. 8B, C). These findings align with those of the ANN model, indicating that a higher proportion of patients with Subtype A exhibited severe disc degeneration, whereas those classified as Subtype B tended to display milder disc degeneration, as represented in the Sankey diagram (Fig. 8C).

### Pathway activity and immune infiltration landscape between two stages of IVDD

**Fig. 9:**
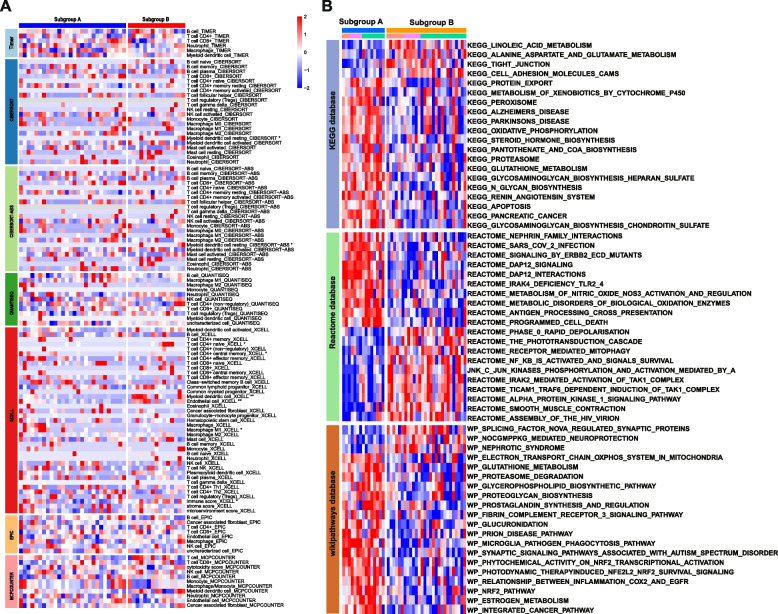
Biological characteristics of different IVDD subtypes. **A** A representative heatmap depicting the differences in the immune landscape in IVDD between Subtype A and Subtype B, as determined using CIBERSORT, TIMER, QUANTISEQ, CIBERSORT-ABS, EPIC, and XCELL algorithms. The bar chart on the right elucidates the correlation between immune cells or stromal cells and their respective subtypes. **B** A heatmap illustrating differential pathway activity between the two subtypes, based on GSVA using the KEGG, Reactome, and WikiPathways databases

To elucidate the functional implications of the hub genes and the underlying mechanisms influencing the progression of IVDD, GSEA was conducted to identify dysregulated biological processes and pathways between the two subgroups. The aim was to determine which biological processes were substantially enriched in one group compared to the other. The results revealed that Subtype B exhibited enrichment in the following processes: cellular response to external stimuli, reactive nutrient deprivation, reactive signaling interleukins (ILs), reactive cell dynamics signaling systems, and reactive metabolic disease signaling (Fig. 8D–H). Conversely, Subtype A showed higher richness in amino acid and derivative metabolism (Fig. 8I). To further investigate potential differences in relevant functions and pathways between these distinct subtypes, analyses were performed using the KEGG, Reactome, and WikiPathways databases to reveal differences in the underlying mechanisms of disease progression between the subtypes. Compared to Subtype B, Subtype A exhibited a remarkable upregulation of inflammatory-related pathways, including apoptosis, the prostaglandin metabolic pathway, pantothenic acid and CoA biosynthesis, the peroxisome pathway, oxidative phosphorylation, as well as the metabolism of nitric oxide and glutathione (Fig. 9A).

Single-cell analysis showed different interactions between NPCs with varying AA metabolic activities and the immune microenvironment. Therefore, it was imperative to investigate the correlation between these subtypes and immune cell populations. To this end, various algorithms, including CIBERSORT, TIMER, QUANTISEQ, CIBERSORT-ABS, EPIC, and XCELL, were employed to assess immune cell infiltration levels across the subtypes. Subtype A exhibited a heightened infiltration of pro-inflammatory immune cells, including M1 macrophages, whereas Subtype B displayed elevated levels of anti-inflammatory immune cell infiltration, including myeloid dendritic cells, CD4 + T cells, and endothelial cells (Fig. 9B).

### Prediction of subtype-specific small molecular compounds and their mechanisms of action

**Fig. 10:**
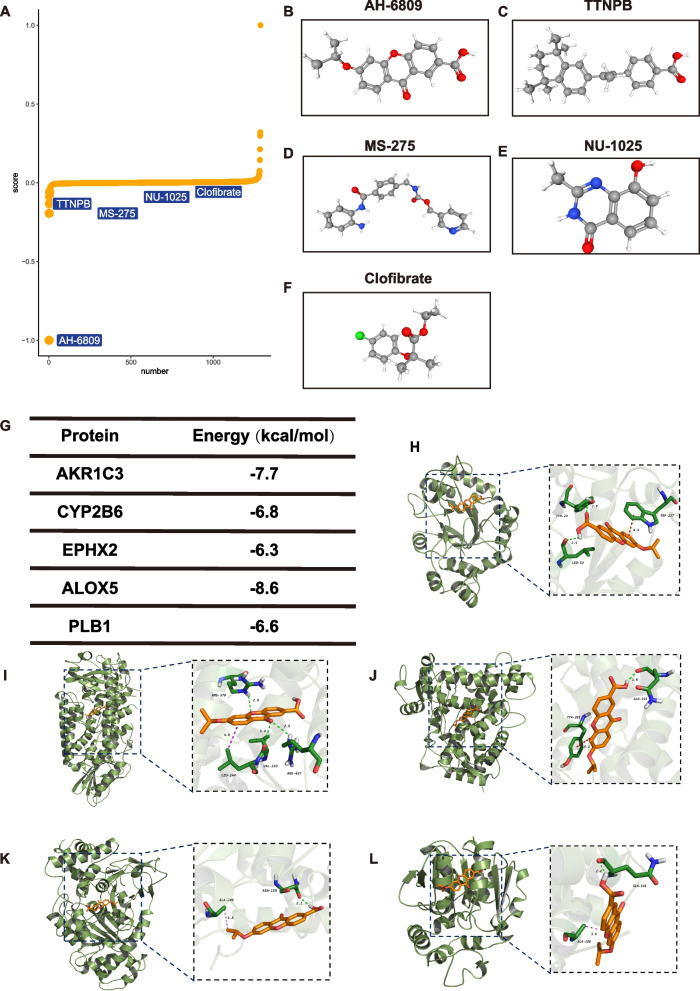
Five small-molecule drugs identified through XSum analyses. **A**–**F** PubChem database displays the molecular structures of the five targeted drugs, including AH6809 (**B**), TTNPB (**C**), MS-275 (**D**), NU-1025 (**E**), and clofibrate (**F**). **G**–**L** Visualization of molecular docking

Subtype A- and Subtype B-specific small molecular compounds were predicted utilizing the XSum algorithm to evaluate their potential as drug candidates against the distinct subtypes. Based on the disease subtyping approach based on the five hub genes, XSum suggested several drugs for the treatment of IVDD, namely AH6809, TTNPB, MS-275, NU1025, and clofibrate. The chemical structures of these drugs are illustrated in Fig. 10A–F.

### Drug–gene interaction and molecular docking analyses of hub genes

Based on the XSum results, molecular docking analysis of AH6809 with AKR1C3, CYP2B6, EPHX2, ALOX5, and PDE1B revealed robust docking of AH6809 with these hub genes (Fig. 10G). The binding affinity between AKR1C3 and AH6809 was -7.7 kcal/mol. In this interaction, the hydroxyl group of TYR at position 24 in AKR1C3 formed a hydrogen bond with the carbonyl oxygen atom of AH6809 at a distance of 2.9 Å. Similarly, the carbonyl group of LEU at position 54 in AKR1C3 formed a hydrogen bond with the hydroxyl group of AH6809 at a distance of 2.5 Å. Additionally, the phenyl ring of TRP at position 227 in AKR1C3 engaged in π–π conjugation with the phenyl ring of AH6809 at a distance of 4.4 Å (Fig. 10H). Supplementary file S2 presents the docking results for AH6809 with the other hub genes (Fig. 10I−L).

### Friends analysis of hub genes

**Fig. 11:**
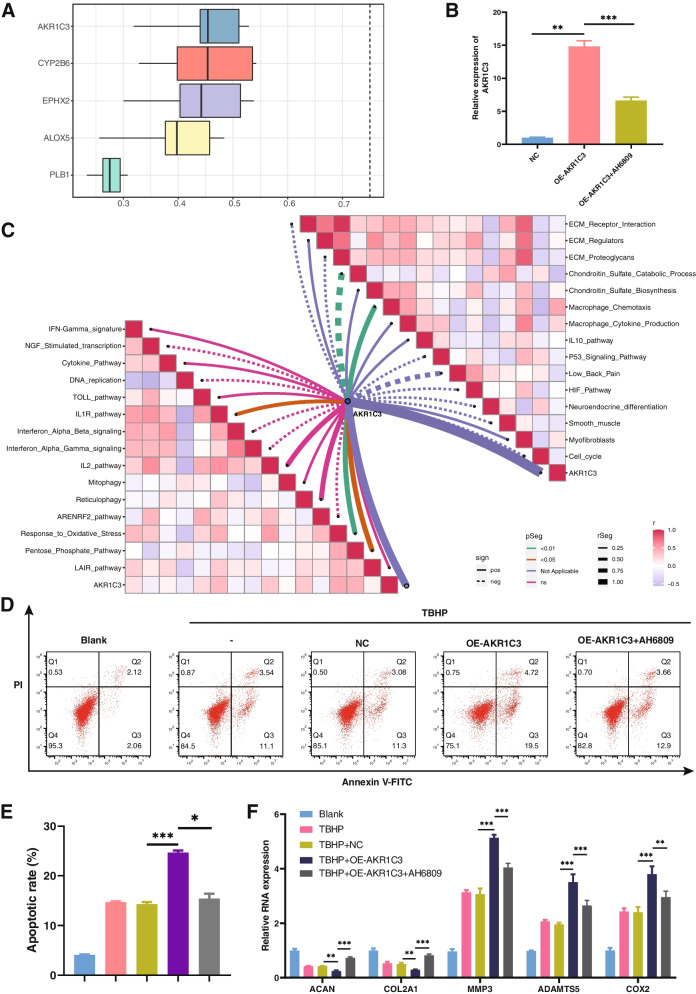
Inhibiting AKR1C3 using AH6809 treats IVDD. **A** A box plot illustrates the functional similarity of the five hub genes, as revealed through Friends analysis. AKR1C3 exhibits the highest degree of correlation with other genes. **B** AKR1C3 expression in NPCs transfected with an NC negative control (empty plasmid) or an AKR1C3 overexpression plasmid, as detected by RT-qPCR. Data are expressed as mean ± SD (*n* = 3) (*, *P* < 0.05; **, *P* < 0.01; ***, *P* < 0.001). **C** Correlation between AKR1C3 and various biological processes. **D**, **E** Apoptosis in NPCs transfected with a control NC plasmid or an AKR1C3 overexpression plasmid, as detected by flow cytometry. Data are expressed as mean ± SD (*n* = 3) (*, *P* < 0.05; **, *P* < 0.01; ***, *P* < 0.001). **F** Changes in gene expression of anabolic markers (ACAN and COL2A1) and catabolic markers (MMP-3, ADAMTS5, and COX2), as determined by RT-qPCR. Data are expressed as mean ± SD (*n* = 3) (*, *P* < 0.05; **, *P* < 0.01; ***, *P* < 0.001)

Using the Friends algorithm, AKR1C3 was found to hold the greatest biological significance among the five hub genes (Fig. 11A). As depicted in Fig. 11C, several pro-inflammatory pathways were significantly negatively associated with AKR1C3, including the IL1R, oxidative stress pathway, pentose phosphate metabolic pathway, and chemotaxis behavior of macrophages.

### AH6809 alleviates IVDD by inhibiting AKR1C3

To date, AKR1C3 has not been reported in association with IVDD. To corroborate the biological alterations resulting from the upregulation of AKR1C3, qRT-PCR was employed to assess the relative efficacy of AKR1C3 overexpression in NPCs (Fig. 11B). To further substantiate this conjecture, overexpression of AKR1C3 was induced in the presence of TBHP. The results indicated that AKR1C3 overexpression enhanced TBHP-induced apoptosis in NPCs (Fig. 11D, E). Subsequent experiments revealed that AKR1C3 overexpression influences NPC proliferation and apoptosis, ECM synthesis and degradation, as well as the release of inflammatory factors by NPCs. RT-qPCR demonstrated an increase in catabolic markers, including MMP-3 and ADAMTS5, coupled with a decrease in anabolic markers, such as ACAN and COL2A1, as AKR1C3 expression levels increased (Fig. 11F). Furthermore, AKR1C3 was found to impact IVDD by modulating the synthesis and metabolism of cyclooxygenase-2 (COX2). These findings collectively establish that AKR1C3, as an integral component of the hub gene network, actively participates in the regulation of IVDD.

Furthermore, given the pronounced affinity between AKR1C3 and AH6809, efforts were initiated to validate the connection between AH6809 and AKR1C3. The results revealed that AH6809 possesses the capacity to alleviate IVDD by suppressing AKR1C3 expression, subsequently influencing apoptosis in NPCs and ECM synthesis. In summary, these results imply that AH6809 holds promise as a potential treatment for IVDD through the inhibition of AKR1C3.

## Discussion

IVDD is widely acknowledged as the primary etiological factor responsible for lower back pain [34]. In clinical practice, the assessment of IVDD prognosis and the formulation of treatment strategies often rely on MRI grading and various pathological parameters [35]. Mild degeneration typically manifests as a reduction in intervertebral disc height and moisture content, accompanied by limited lower back pain symptoms or the absence of compressed nerve root involvement [7]. In such cases, clinicians recommend non-surgical interventions, encompassing physical therapy and NSAIDs, to alleviate patient discomfort [36]. Conversely, severe degeneration is characterized by a significant reduction in intervertebral disc height, intervertebral space narrowing, and conspicuous lower back pain symptoms or compressed nerve roots. In these instances, surgical intervention becomes the only viable clinical recourse, encompassing procedures such as intervertebral disc removal, laminectomy, facetectomy, and artificial disc replacement [37, 38]. Therefore, timely diagnosis and intervention in IVDD can offer distinct advantages by mitigating the necessity for surgical interventions and enhancing healthcare resource utilization, thereby yielding substantial clinical and socio-economic benefits.

The etiology of IVDD is considered multifactorial, with factors such as smoking, aging, infection, genetic susceptibility, and abnormal biomechanical loading [39, 40]. Importantly, irrespective of the initial factors, IVDD degeneration is attributed to the secretion of pro-inflammatory molecules by NPCs and peripheral immune cells [33, 41]. AA and its metabolites assume a pivotal role in the inflammatory response associated with IVDD. Compounds such as prostaglandin E2 (PGE2) and leukotriene B4, generated through AA metabolism, can promote inflammation and intervertebral disc degradation, while agents inhibiting AA metabolism, such as NSAIDs and COX2 inhibitors, can ameliorate the inflammatory response in IVDD [42, 43]. Furthermore, heightened AA metabolic pathway activity expedites the IVDD process [6]. Therefore, it is imperative to conduct further research on biomarkers and pathological mechanisms associated with AA metabolism in IVDD to advance and refine personalized treatment strategies.

Using scRNA-seq data, the microenvironmental landscape of NP was delineated. NPCs were found to constitute more than 90% of the tissue and represent the predominant cellular component. Within this context, Noto play a pivotal role in preserving NPCs and are closely associated with their repair mechanisms. Notably, Noto were observed in mild-IVDD cases but were nearly absent in severe IVDD. Moreover, an increase in neutrophils and macrophages was observed in severely degenerated NP tissue, implying that inflammatory activation may be a notable contributor to the acceleration of IVDD. These findings underscore the potential benefits of early intervention in IVDD to mitigate inflammatory responses, reduce Noto apoptosis, and facilitate the regeneration of NPCs.

Furthermore, AA metabolism within the intervertebral disc microenvironment was analyzed. The findings indicate that as disc degeneration progresses, AA metabolism becomes increasingly active. Moreover, notable variations in AA metabolism were observed among NPCs at varying degeneration stages. To explore the heterogeneity in the regulation of NPCs by AA metabolism, the harmony integration algorithm was employed to categorize cells into distinct subtypes. Pseudotime analysis further illuminated that heightened AA metabolic activity correlates with a proclivity toward degenerative development. These findings underscore the potential for mitigating IVDD by regulating AA metabolism, identifying viable therapeutic targets, and designing corresponding pharmaceutical interventions.

Furthermore, NPCs exhibiting varying levels of AA metabolism displayed divergent communication patterns with neighboring cells within the microenvironment. The communication dynamics were compared between NPCs operating at distinct metabolic levels and other cell types within the intervertebral disc NP tissue. This analysis encompassed an examination of receptor interactions and signaling pathways. The findings underscore that NPCs with heightened AA metabolism are more likely to modulate neighboring microenvironmental cells. Conversely, NPCs with diminished AA metabolism tend to be subject to regulation by surrounding microenvironmental cells. This regulation is associated with processes such as cell proliferation, ECM formation, and anti-aging mechanisms. These findings suggest that the manipulation of AA metabolism in NPCs may hold promise as a means to influence the biological functions of the microenvironment. Such an approach could potentially enhance the anti-apoptotic and regenerative capabilities of NPCs.

Subsequently, AI technology was leveraged innovatively to discern stable and robust prognostic biomarkers for IVDD. Initially, a genomics-driven predictive model was developed for the early diagnosis of IVDD. Compared to CRP, the model showcases superior efficacy in gauging both the extent of disc degeneration and the inflammatory activity in patients. Additionally, the model enables patient stratification and facilitates tailored drug interventions. These findings offer a more scientifically informed approach to clinical diagnosis and treatment, furnishing biological substantiation and rationales for personalized healthcare in the context of IVDD.

Five hub genes were found to be crucial to the pathogenesis of IVDD, playing a significant role in its treatment. *AKR1C3*, a member of the aldo–keto reductase family, is involved in multiple metabolic pathways and signaling cascades. It catalyzes the direct reduction of PGH2 to PGF2α, thereby promoting the release of inflammatory mediators, which in turn intensify the magnitude and duration of the inflammatory response [44]. *ALOX5*, a key rate-limiting enzyme in AA metabolism, is responsible for catalyzing the conversion of 5-hydroperoxyeicosatetraenoic acid to LTA4, thus contributing to the occurrence and progression of inflammation and degenerative diseases [45]. *CYP2B6*, a member of the cytochrome P450 family, actively regulates the intensity and duration of inflammatory reactions. Its expression is modulated by various inflammation-related signaling pathways, such as nuclear factor-kappa β and cytokines [46, 47]. Research has indicated a significant upregulation of *CYP2B6* expression under inflammatory conditions, facilitating the regulation of inflammatory responses through metabolite generation and modulation of signaling pathways [48]. *EPHX2*, encoding a membrane esterase, catalyzes the hydrolysis of epoxy prostaglandins, thus modulating the equilibrium between *PGE2* and prostaglandin D2, among others. Numerous studies have established *EPHX2*'s inhibitory impact on the secretion of inflammatory cytokines and the gene expression of COX2, thereby manifesting pro-inflammatory effects [49]. *PLB1*, a member of the phospholipase family, primarily facilitates the hydrolysis of phosphatidic acid esters, thereby regulating inflammation's initiation and progression through its involvement in prostaglandin and leukotriene production [50]. Based on the stratification of patients with IVDD based on these five genes, medications may be tailored to the patients and facilitate the amelioration of IVDD and the reversal of disease onset.

To further elucidate the biological functions of the five hub genes, PCA was conducted based on their expression levels. This analysis facilitated the categorization of all samples into two distinct subtypes: Subtype B, characterized by milder disc degeneration, and Subtype A, characterized by more severe disc degeneration. Examination of immune cell infiltration patterns underscored the unique immune cell profiles and immune functions associated with these molecular subtypes. Comparing Subtype A to Subtype B, comparable ratios of cellular infiltration and shared molecular characteristics, including myeloid dendritic cells, CD4 + T cells, and endothelial cells, were observed, which suggests an increased prevalence of anti-inflammatory cells in Subtype B. Conversely, Subtype A exhibited a heightened proportion of M1 macrophages, indicative of more pronounced inflammatory activity within this subtype. GSVA revealed that the disparities between these subtypes were associated with inflammation and ECM regulation. Concurrently, the differential expression of inflammation-related genes between the subtypes underscored higher levels of pro-inflammatory factors in Subtype A relative to Subtype B.

Given the intimate relationship between subtypes and underlying biological distinctions, the evaluation of small-molecule drugs assumes translational significance within clinical settings. Through XSum analysis, five small molecular drugs (AH6809, TTNPB, MS-275, NU1025, and clorfibrate) were identified for potential use in the early reversal of IVDD. The chemical structure of AH6809 comprises a benzene ring and an imidazole ring, with a molecular formula of C_17_H_14_N=_2_O_2_. This small-molecule compound serves as a PGE2 receptor antagonist and has exhibited efficacy in the treatment of rheumatoid arthritis, gout, and inflammatory bowel disease [51]. TTNPB functions by inhibiting the release of inflammatory factors by binding to the retinoic acid receptor or vitamin D receptor. Additionally, it possesses the ability to attenuate oxidative stress and impart anti-aging effects on cells. The pharmacological activity of TTNPB aligns with the mechanisms underlying IVDD, rendering it potentially valuable in the treatment of this condition [52]. MS-275 is an HDAC inhibitor capable of suppressing the expression of inflammation-related genes by inhibiting HDAC enzyme activity, thus mitigating the inflammatory response [53, 54]. NU1025 is a potent Poly (ADP-ribose) polymerase (PARP) inhibitor extensively studied and applied in cancer treatment [55, 56]. However, it is important to highlight that PARP inhibitors effectively mitigate NAD + and ATP depletion, subsequently reducing cellular oxidative stress levels and providing cellular protection against oxidative damage [56]. Clorfibrate, formerly employed for high cholesterol and triglyceride management, falls within the category of fibric acid derivatives [57, 58]. A few studies suggest that fibric acid derivatives may possess anti-inflammatory and antioxidant properties, but further investigation is required to elucidate the relationship between clorfibrate and inflammation [59].

To validate the findings, the influence of AKR1C3 on NPCs was examined. The examination revealed that heightened AKR1C3 expression correlates with elevated instances of apoptosis, ECM degradation, and accelerated IVDD. Molecular docking results indicated a promising interaction between AH6809 and AKR1C3, implying the potential of AH6809 to inhibit NPC apoptosis and ECM degradation. Consequently, AH6809 emerges as a prospective pharmaceutical agent for the treatment of IVDD. These assertions were validated through flow cytometry and RT-PCR.

## Strengths and limitations

By harnessing machine learning and ANNs, biomarkers associated with the AA metabolism pathway were identified, demonstrating exceptional efficacy in predicting the progression of IVDD in patients. These biomarkers hold substantial promise as potential biological indicators for the clinical diagnosis of IVDD. The findings underscore the capacity of AI and genomics to identify high-risk individuals at an early stage, thus facilitating targeted preventive interventions through proactive monitoring and management. For instance, in the context of early-stage IVDD, pharmacological interventions, such as the five drugs identified in this study, can be employed for early intervention, mitigating inflammation, and achieving the objective of prevention. Additionally, patients with early-stage IVDD may derive benefits from the utilization of lumbar support belts or participation in rehabilitative exercises designed to enhance the functionality of lumbar and back muscles, thereby facilitating early prevention, precise diagnosis, and alleviating the economic burden on both patients and society. Therefore, the disease stratification predictive model, based on the AA metabolism pathway, holds substantial promise as a prospective molecular biomarker.

Although this study has yielded promising results, there are several limitations that require attention. Firstly, to validate the study findings and enhance their generalizability, larger-scale and multi-center studies are imperative. Employing such study designs will enable the presentation of more compelling evidence to corroborate the study findings. Additionally, conducting in vitro drug testing and clinical trials is crucial to corroborate the therapeutic effects of various small molecules on the progression of IVDD. These validations will establish a robust foundation for this study and facilitate the application of the study findings into the realm of personalized medicine for managing IVDD progression.

## Conclusion

By elucidating the role of AA metabolism in IVDD, this study seeks to advance the understanding of this complex condition. Through the identification of robust biomarkers and the development of predictive models, this study aim to improve early detection, risk assessment, and treatment decision-making for IVDD patients. Such endeavors hold great promise for optimizing patient outcomes, reducing the reliance on surgical interventions, and paving the way for more effective, personalized approaches in the management of IVDD.

### Supplementary Information

**Additional file 1.**

**Additional file 2.**

## Data Availability

The datasets generated and/or analyzed during the current study are available in the GEO database: GSE176205: https://www.ncbi.nlm.nih.gov/geo/query/acc.cgi?acc=GSE176205; GSE67567: https://www.ncbi.nlm.nih.gov/geo/query/acc.cgi?acc=GSE67567; GSE205535: https://www.ncbi.nlm.nih.gov/geo/query/acc.cgi?acc=GSE205535; GSE70362: https://www.ncbi.nlm.nih.gov/geo/query/acc.cgi?acc=GSE70362.

## Abbreviations

IVDD: Intervertebral discs degeneration
NP: Nucleus pulposus
NPCs: Nucleus pulposus cells
AA: Arachidonic acid
ANN: Artificial neural network
NSAIDs: Nonsteroidal anti-inflammatory drugs
scRNA-seq: Single-cell sequencing
MRI: Magnetic resonance imaging
AI: Artificial intelligence
AKR1C3: Aldo-Keto Reductase Family 1 Member C3
ALOX5: Arachidonate 5-Lipoxygenase
CYP2B6: Cytochrome P450 Family 2 Subfamily B Member 6
EPHX2: Epoxide Hydrolase 2
PLB1: Phospholipase B1
ACAN: Aggrecan
COL2A1: Collagen Type II Alpha 1
RT-qPCR: Real-time reverse transcription polymerase chain reaction
