# Supplementary material for: Role of arachidonic acid metabolism in intervertebral disc degeneration: identification of potential biomarkers and therapeutic targets via multi-omics analysis and artificial intelligence strategies
Source: Lipids Health Dis. 2023 Nov 25;22:204. doi: 10.1186/s12944-023-01962-5 (PMC10675942; doi:10.1186/s12944-023-01962-5)

1. CYP2B6

The binding affinity between CYP2B6 and AH6809 is -6.8 kcal/mol. In this interaction, the carbonyl group of GLN at position 193 in CYP2B6 forms a hydrogen bond with the hydroxyl group of AH6809 at a distance of 1.9 Å. Similarly, the phenyl ring of TYR at position 203 in CYP2B6 forms a π-π conjugation with the phenyl ring of AH6809 at a distance of 6.1 Å.


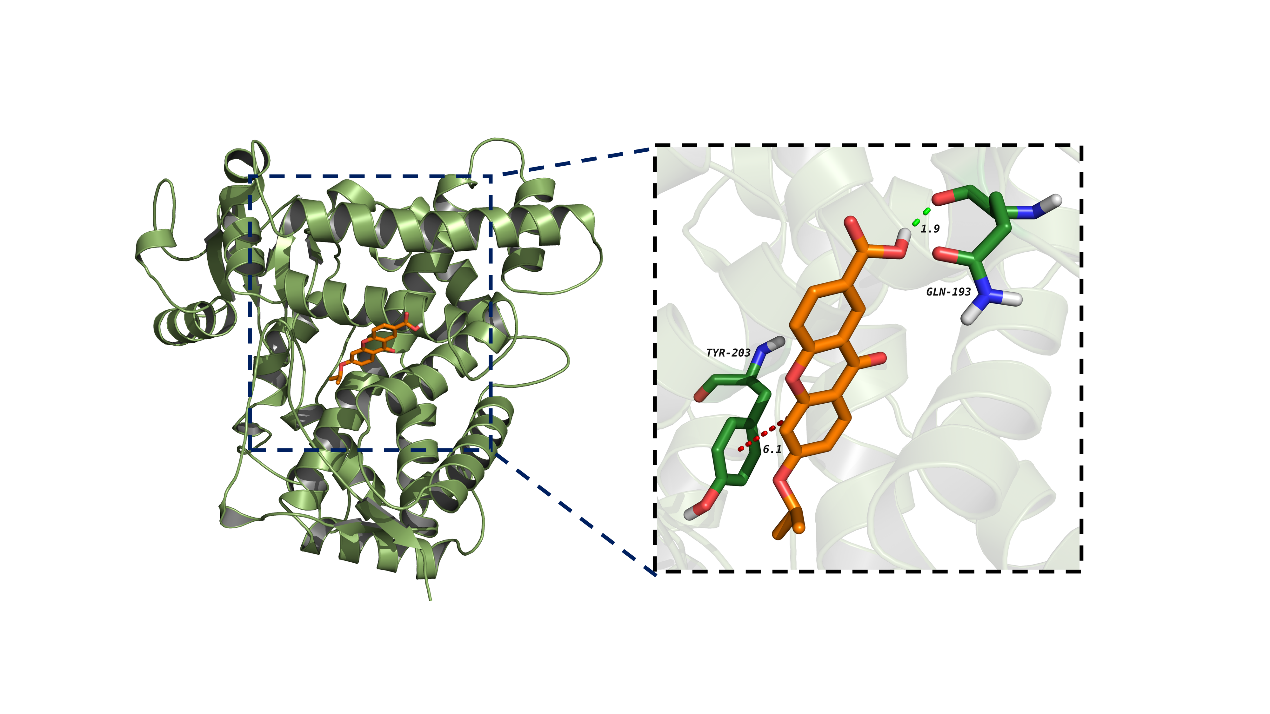


2. EPHX2

The binding affinity between EPHX2 and AH6809 is -6.3 kcal/mol. In this interaction, the carbonyl group of GLN at position 536 in EPHX2 forms a hydrogen bond with the hydroxyl group of AH6809 at a distance of 2.4 Å. Similarly, the aromatic structure of ALA at position 280 in CYP2B6 forms a pi-sigma conjugation with AH6809 at a distance of 3.9 Å.


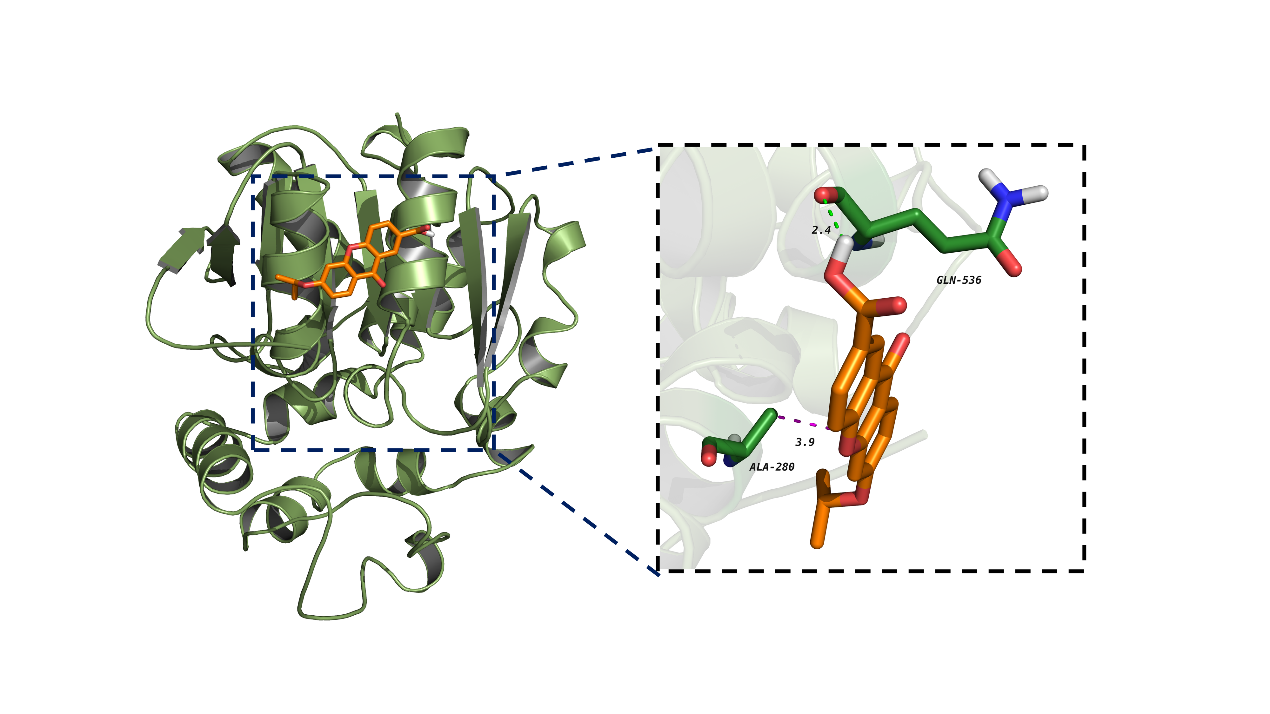


3. ALOX5

The affinity of the ALOX5 and AH6809 binding is -8.6 kcal/mol. In this interaction, a hydrogen bond is formed at a distance of 3.4 Å between the carbonyl group of VAL at position 243 of ALOX5 and the carbonyl group of AH6809. Similarly, at a distance of 4.0 Å, a pi-sigma conjugation is formed between LEU at position 244 of ALOX5 and the benzene ring of AH6809. A hydrogen bond is also formed between the amino hydrogen of ARG at position 370 and the oxygen atom of the ether bond of AH6809, with an interaction distance of 2.6 Å. Another hydrogen bond is established between the amino hydrogen of ARG at position 457 and the carbonyl group of AH6809, with an interaction distance of 2.9 Å.


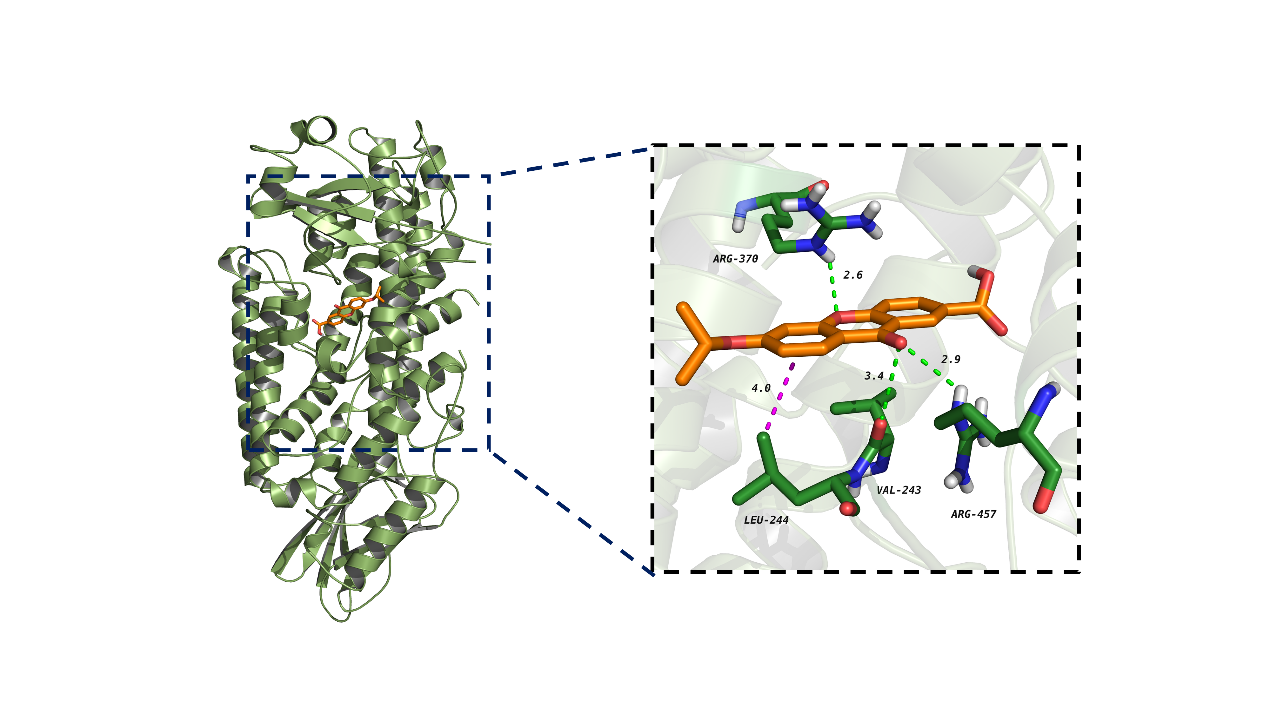


4. PLB1

The binding affinity between PLB1 and AH6809 is -6.6 kcal/mol. In this interaction, the carbonyl group of ASN at position 159 of PLB1 forms a hydrogen bond with the hydroxyl group of AH6809 at a distance of 2.1 Å. Similarly, the ALA at position 249 of PLB1 forms an alkyl conjugation with AH6809 at a distance of 3.8 Å.


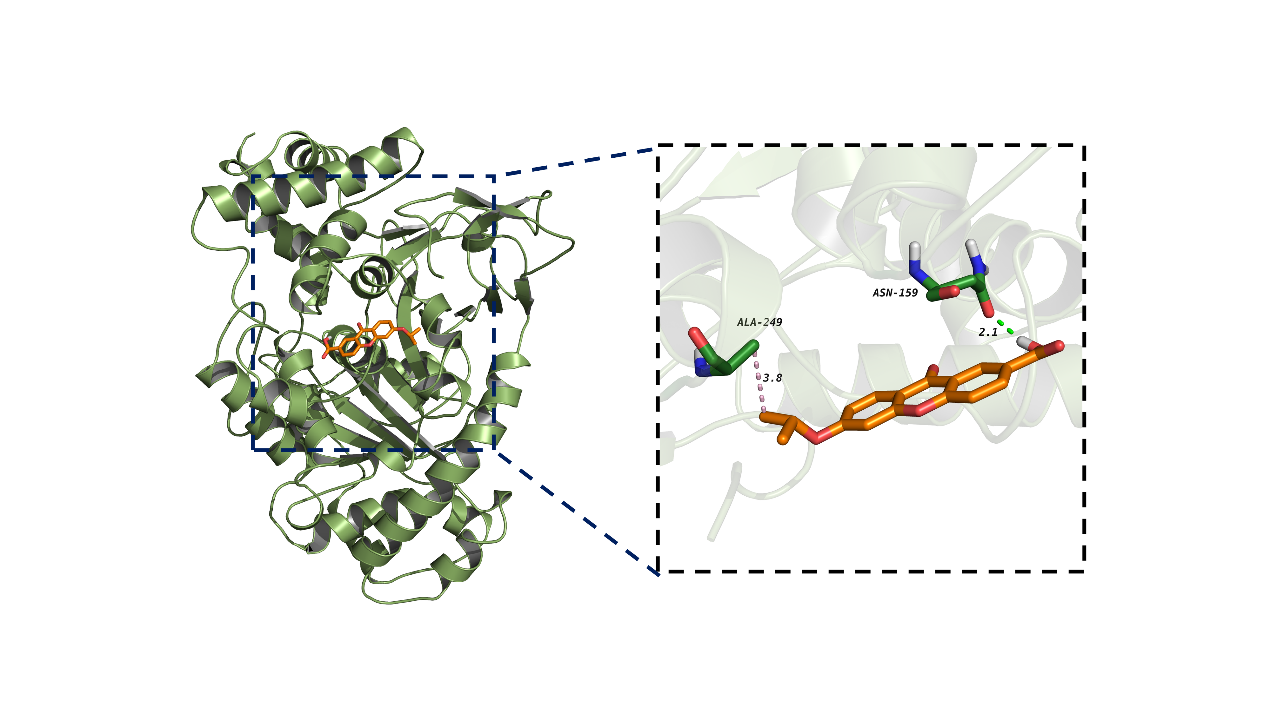

Supplement: Supplementary file 2 — Additional file 2. [file 12944_2023_1962_MOESM2_ESM.docx]
